# The stromal vascular fraction mitigates radiation-induced gastrointestinal syndrome in mice

**DOI:** 10.1186/s13287-021-02373-y

**Published:** 2021-05-29

**Authors:** Lydia Bensemmane, Claire Squiban, Christelle Demarquay, Noëlle Mathieu, Marc Benderitter, Bernard Le Guen, Fabien Milliat, Christine Linard

**Affiliations:** 1 Institute of Radiological Protection and Nuclear Safety, Laboratory of Medical Radiobiology, Fontenay-aux-Roses, France; 2 Electricité de France, Cap Ampère, Saint-Denis, France

**Keywords:** Stromal vascular fraction, Intestine, Irradiation, Regeneration

## Abstract

**Background:**

The intestine is particularly sensitive to moderate-high radiation dose and the development of gastrointestinal syndrome (GIS) leads to the rapid loss of intestinal mucosal integrity, resulting in bacterial infiltration, sepsis that comprise patient survival. There is an urgent need for effective and rapid therapeutic countermeasures. The stromal vascular fraction (SVF) derived from adipose tissue is an easily accessible source of cells with angiogenic, anti-inflammatory and regenerative properties. We studied the therapeutic impact of SVF and its action on the intestinal stem cell compartment.

**Methods:**

Mice exposed to the abdominal radiation (18 Gy) received a single intravenous injection of stromal vascular fraction (SVF) (2.5 × 10^6^ cells), obtained by enzymatic digestion of inguinal fat tissue, on the day of irradiation. Mortality was evaluated as well as intestinal regeneration by histological analyses and absorption function.

**Results:**

The SVF treatment limited the weight loss of the mice and inhibited the intestinal permeability and mortality after abdominal irradiation. Histological analyses showed that SVF treatment stimulated the regeneration of the epithelium by promoting numerous enlarged hyperproliferative zones. SVF restored CD24^+^/lysozyme^−^ and Paneth cell populations in the ISC compartment with the presence of Paneth Ki67^+^ cells. SVF has an anti-inflammatory effect by repressing pro-inflammatory cytokines, increasing M2 macrophages in the ileum and anti-inflammatory monocyte subtypes CD11b^+^Ly6c^low^CX3CR1^high^ in the spleen.

**Conclusions:**

Through the pleiotropic effects that contribute to limiting radiation-induced lethality, SVF opens up attractive prospects for the treatment of emergency GIS.

**Supplementary Information:**

The online version contains supplementary material available at 10.1186/s13287-021-02373-y.

## Background

Accidental or intentional exposure to high doses of ionising radiation can have serious health consequences for the exposed persons and can potentially affect many people at the same time. Irradiation of large volumes at medium to high radiation doses has serious effects on normal, fast-renewing tissues, and all these effects are grouped together under the acute radiation syndrome (ARS). The speed at which the first symptoms appear depends on the rate of renewal of the cells making up the damaged tissue, but also on the radiosensitivity of the stem cells. The gastrointestinal (GI) tract is particularly sensitive to irradiation and lethality resulting from GI tract failure, which is the primary endpoint of ARS. A dose greater than 10 Gy in total body irradiation (TBI) leads to diarrheoa, dehydration, sepsis and intestinal haemorrhage with mortality within 7–10 days after exposure.

Normal homeostasis of the intestinal epithelium is maintained by a complex cell replacement process. This renewal is initiated by intestinal stem cells (ISCs), notably the Lgr5^+^ cells located at the base of the crypts [1]. These stem cells divide, migrate and become Lgr5 progenitors or transit-amplifying progenitor (TA) cells committed to differentiation. Differentiated TA cells generate villi composed of intestinal epithelial cells (IECS). Lgr5^+^ cells are flanked on both sides by secretory Paneth cells. In addition to their antimicrobial role, these cells have played an essential niche role in supporting Lgr5^+^ cells, both through cell-cell contact and by secreting the factors necessary to maintain the gut stem cell niche [2].

Radiation-induced gastrointestinal syndrome (GIS) is due in part to the destruction of intestinal stem cells (ISCs) in the crypt [3], which are radiosensitive. The crypt becomes “non-viable” and dies out within 48 h. However, one or more surviving ISCs can rapidly proliferate to regenerate the crypt within 72–96 h. Therefore, the repopulation of clonogenic proliferating progenitor cells by the ISCs plays a key role in the fate of the crypt. A damaged intestinal epithelium promotes the influx of bacteria into the bloodstream leading to sepsis and death [4]. Considering the logistical barrier and urgency delay within the first 24 h after exposure for treatment of the victim, in large casualty settings, there is a concrete need for therapeutic measures that can be effective even if started days after the accidental radiation exposure. Currently, there are very few drugs known to mitigate the effects of radiation. In part of an artificial analogue of flagellin, which has improved gut stem cell survival after whole body irradiation in mice [5] and in a non-human primate [6], it may have mitigating pharmacological treatment potential, but there is no medical countermeasure for gastrointestinal treatment in humans.

Studies have shown that cell therapies restore the niche of intestinal stem cells after irradiation [7] and that mesenchymal stem cell (MSC) administration can improve survival, restore intestinal structure and function and increase epithelial cell proliferation [8–11]. However, the culture time required to amplify enough cells remains an obstacle to their use in the emergency treatment of gastrointestinal syndrome (GIS).

It has been recently demonstrated that adipose tissue can be an important source of cell progenitors, easily accessible and extremely rapid. Adipose tissue-derived stem cells (ASCs) share many properties with bone marrow mesenchymal stem cells. They have the potential to differentiate into multiple cell lineages and have angiogenic, anti-apoptotic, immunomodulatory and regenerative properties [12]. ASCs have self-renewal properties and are localised in the stromal vascular fraction (SVF) obtained from enzymatically digested adipose tissue. SVF constitutes a vascular niche with an important source of endothelial progenitors, endothelial cells and pericytes, thus contributing to vessel remodeling and growth [13]. In addition, SVF contains various immune cells, including monocytes and macrophages that perform anti-inflammatory functions [14]. The mechanisms through which SVF regenerates tissue remain inconclusive, but the literature suggests a contribution by paracrine effects, with crosstalk between SVF components and the host leading to repair and healing [12]. Currently, the therapeutic use of SVF is expanding in some clinical trials of multiple pathologies [12], particularly for refractory Crohn’s fistula [15].

In this study, we evaluated the therapeutic efficacy of SVF in a GIS model and examined the impact of SVF on the niche compartment of intestinal stem cells and the impact on the inflammatory process.

## Methods

### Mice

Male C57BL/6JRj mice were purchased from Janvier (Le Genest Saint Isle, France) and the animals were housed in the IRSN animal facilities, which are accredited by the French Ministry of Agriculture for performing experiments on rodents. The animal experiments were performed in compliance with French and European regulations on the protection of animals used for scientific purposes (EC Directive 2010/63/EU and French Decree 2013–118). All experiments were approved by the Ethics Committee #81 and authorised by the French Ministry of Research (under the reference APAFIS#10434-2017062814437026-v1). In this study, the experimental protocol was in line with the standard support treatment recommended for the patient presenting an acute radiation syndrome [16]. Thus, the antibiotic Avemix (8g/L) will be associated with all treatments and during the experimentation.

### Irradiation procedure

Eight-week-old C57BL/6JRj mice were irradiated under anaesthesia (with a continuous flow 1.5% isoflurane in oxygen) on a medical linear accelerator (Elekta synergy®) delivering 4MVp X-rays (mean photon energy about 1.3 MeV). Reference dosimetry measurements were performed using a 0.125 cc cylindrical ionisation chamber calibrated in dose to water in a mouse equivalent tissue phantom placed on a plexiglass support. A dose rate about 2.5 Gy/min in dose to water was used. A localised 2-cm larger abdominal irradiation window containing intestine was chosen to limit the upper thorax, head and neck and lower and upper extremity exposure. The mice were exposed to 16, 18, 20 and 22 Gy. The dose of 18 Gy was further selected as the sublethal optimal irradiation dose for SVF-related effects. The uncertainty of the dose rate measurement was about 5% at k = 2.

### Isolation of SVF

Stromal vascular fraction (SVF) cells were isolated from inguinal fat pad adipose tissue samples of 12-week-old C57BL/6JRj mouse donors. From the modified methods [17], the fat pads were excised, finely cut and incubated in a digestion medium containing 0.1% type I collagenase (Sigma-Aldrich, France) and 1% penicillin-streptomycin in MEM-α milieu by gentle shaking for 20 min at 37°C. The samples were briefly mechanically disrupted using the gentleMACS Dissociator (Miltenyi Biotec) and then digested again in MEM-α 0.1% type I collagenase medium followed by mechanical disruption using the gentleMACS Dissociator. To remove mature adipocytes and undigested tissue, the cell suspension was filtered sequentially through 100- and 70-μm cell strainers and centrifuged (400*g*, 10 min) to spin down stromal vascular fraction cell pellets. The pellets were resuspended in phosphate-buffered saline (PBS) for i.v. injection.

### Histology

#### Microcolony stem cell assay

Freshly isolated ileal tissue was excised, flushed with cold phosphate-buffered saline and fixed in 4% paraformaldehyde and embedded in paraffin. At day 3.5, ileal sections were stained using haematoxylin, eosin and Safran (HES). The sublethal dose was determined by the number of regenerating crypts along the ileum with the presence of 10% of the crypts by unit of length of intestine containing at least 8–10 cells at day 3.5 after irradiation [18].

#### Immunohistochemical analysis

Sections 5 μm in thickness were de-paraffinised and re-hydrated. A pretreatment method using heat-induced epitope retrieval was used, and the nonspecific binding was blocked with a protein blocker (DakoCytomation, Trappe, France) for 30 min at room temperature (RT). The sections were then incubated with the following primary antibodies against Ki67 (ab15580; Abcam), EpCam (clone G8.8; Biolegend), α-SMA (ab21027; Abcam), CD34 (RAM34, eBioscience), GP38 (MA615113; Thermo Fisher), CD24 (ab64064; Abcam), Lysozyme (ab108508; Abcam), Muc2 (Thermo Fisher) and ZO-1 (Invitrogen) antibodies.

The co-labelling with antibodies from the same species was performed using Opal™ Multiplex IHC kits (Akoya). After incubation with the primary antibodies, the slides were washed in PBS-T three times and probed with appropriated fluorescence-conjugated secondary antibodies for 1 h at room temperature. After three washings in PBS, the cell nuclei were counterstained by Vectashield mounting medium with DAPI (Vector).

#### TUNEL assay

A TUNEL assay was performed using an In Situ Cell Death Detection Kit (Roche Diagnostics, France) following the manufacturer’s instructions. Intestinal tissue sections were incubated with a reaction mixture of terminal deoxynucleotidyl transferase (TdT) and fluorescein (FITC)-labelled precursor in a cacodylate-based buffer for 1 h at 37 °C, then rinsed three times with 0.05% Tween-20 in PBS and mounted under Vectashield mounting medium with DAPI (Vector). The apoptotic rate in the crypts and villis cells was quantified by counting the number of apoptotic cells per unit area of the section.

### Intestinal permeability in vivo

In vivo intestinal permeability measurement was assessed at 7 days following irradiation, using 4000 Da FITC-dextran (Sigma-Aldrich, France). In short, FITC-dextran (0.60 mg/g body weight) was administrated by gavage and 4 h after the mice were euthanised and plasma obtained by cardiac puncture. Standard curves were obtained by diluting the FITC-dextran in the PBS-diluted plasma to determine the serum levels of FITC-dextran in the different treatment groups. The FITC concentration in the plasma was then measured with a microplate Luminometer (Mithras LB940, Berthold) at an excitation wavelength of 485 nm and an emission wavelength of 520 nm.

### Splenic cell isolation

To isolate the splenic cells, the spleen was smashed with a syringe plunger and the cells were collected in PBS buffer followed by passage through 40-μm cell strainers and centrifuged (400×*g*, 10 min). The red blood cells were lysed with ACK lysis buffer (Thermo Fisher Scientific, France).

### Flow cytometric analysis

Flow cytometry was performed using the BD FACSCanto II and analysed with FlowJo software (Tree Star, Ashland, OR). Dead cells were excluded through Fixable Viability Dye eFluor staining (eBioscience). Nonspecific antibody binding was blocked with an anti-CD16/32 (Mouse Fcγ block clone 2.4G2). The cells were incubated for 30 min in 100 μl PBS with conjugated antibodies (BD Biosciences, France): anti CD45 (V500 conjugated; clone 30-F11), CD11b (BV421 conjugated; clone M1/70), anti CD3 (FITC conjugated; clone 145-2C11), anti CD45R/B220 (PE-Cy7 conjugated; clone RA3-6B2), anti Ly6C (FITC conjugated; clone AL-21), anti CD31 (PE conjugated; clone MEC13.3), anti CD34 (Alexa Fluor 647 conjugated; clone RAM34), anti CD146 (FITC conjugated; clone ME-9F1), anti CD29 (PE conjugated; clone HM B1.1), anti Sca-1 (PE-Cy7 conjugated; clone D7), anti CD44 (PE-Cy5.5 conjugated; clone IM7), anti CD105 (Alexa Fluor 647 conjugated; clone MJ7/18), anti SSEA (BV421 conjugated; clone MC631), anti CX3CR1 from Biolegend (France) (PE-Cy7 conjugated; clone SA011F11), anti CCR2 (Alexa Fluor 647 conjugated; clone SA203G11) and CD90 from southern Biotech (FITC conjugated; clone SA203G11). Isotype-matched antibodies (BD Biosciences) were used for control staining. All antibodies were used at 1:100 dilution. The concentration of cell suspensions was adjusted to 1 × 10^6^ cells per 100 μl.

### Spreading assay and immunostaining

Spleen mononuclear cells MNCs isolated from all the experimental groups were resuspended in RPMI 1640 medium containing 10% FBS and 1% penicillin/streptomycin and seeded on round 12-mm diameter coverslips in a 24-well plate coated with 5μg/ml recombinant mouse VCAM-1 (R&D Systems) for 2 h at 37°C. After two washes with PBS, the cells were fixed with 4% paraformaldehyde for 10 min at +4°C. The cells were rinsed three times with PBS and then incubated in PBS/0.1% Triton X-100 (Sigma-Aldrich) for 10 min. After three rinses with PBS and incubation with 2% BSA to block nonspecific staining, the cells were stained with 0.165 μM Alexa Fluor 546 Phalloidin (Thermo Fisher Scientific, France) for 60 min at 37°C. The primary antibody anti-CX3CR1 (Abcam) was used for immunostaining. After the incubation, slides were washed with PBS-T and probed with fluorescence-conjugated secondary antibodies for 1 h at RT. The cell nuclei were counterstained by Vectashield mounting medium with DAPI (Vector). Cells with flat morphology or lamellipodia were positively scored for spreading.

### Monocyte/macrophage polarisation

The spleen cells were washed three times and cultured in a 12-well tissue culture in RPMI-1640 supplemented with 10% FBS and antibiotics (penicillin/streptomycin, Invitrogen) and 100 ng/ml of M-CSF (R&D Systems, France) at 37°C in a humidified atmosphere containing 5% CO_2_ for 7 days to induce full macrophage differentiation and maturation. Macrophage polarisation was obtained by removing the culture medium and culturing cells for an additional 24 h in RPMI-1640 supplemented with 10% FBS and antibiotics and 100 ng/ml LPS (derived from *E. coli* 0111:B4, Invivogen) plus 20 ng/ml IFN-γ (for M1 polarisation, M(LPS-IFN-γ)) or 20 ng/ml IL-4 (for M2 polarisation, M(IL-4)) (R&D Systems). The cells were harvested for RT-qPCR analysis.

### Real-time PCR analysis

Total RNA was extracted from the ileum and spleen cells with the RNeasy Mini kit (Qiagen), and cDNA was prepared with the SuperScript RT Reagent Kit (Applied Biosystems). Real-time PCR was performed on an ABI Prism 7900 Sequence Detection System. All Taqman primers and probes came from Life Technologies (France) (Supplemental table). The data were analysed using the 2^−ΔΔCt^ method, with normalisation to the Ct of the glyceraldehyde 3-phosphate dehydrogenase (GAPDH) housekeeping gene.

### Statistics

The data are expressed as the mean ± SEM. We used one-way or two-way analyses of variance (ANOVA) and then a Bonferroni post-test to determine the significance of the differences. *p* values less than 0.05 were considered statistically significant.

## Results

### Characteristics of radiation-induced GIS after abdominal irradiation

**Fig. 1:**
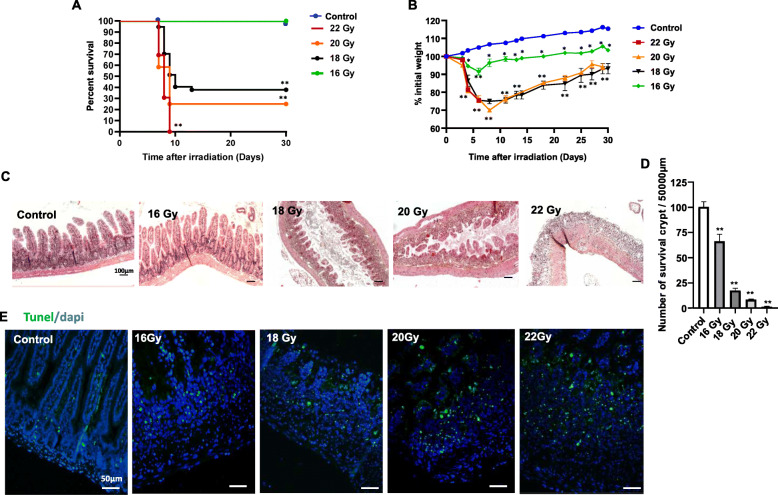
Identification of irradiation dose inducing GIS. **a** Kaplan-Meier survival analysis of 16, 18, 20 and 22 Gy abdominal irradiated mice (n = 10 per group). **b** Weight loss changes for 30 days of mice exposed to 16, 18, 20 and 22 Gy abdominal irradiation (n = 10 per group). **c** Response of the small intestine to 16, 18, 20 and 22 Gy abdominal irradiation at day 3.5. Representative HES staining of the ileum showing structural modification. Scale bar 100 μm. **d** Irradiation dose-dependent regenerative crypts. The plots illustrate the number of surviving regenerative crypts present by unit length in a small intestine 3.5 days post-irradiation. The data are represented by mean ± SEM (n = 4–5), p values were calculated by one-way or two-way analyses (for weight loss changes) of variance (ANOVA) with Bonferroni correction; *p < 0.01; **p < 0.001 compared with the control group. **e** Tunel staining. Representative images of ileal sections at 3.5 days post-irradiation demonstrating an increase of Tunel-positive cells at the crypt base with the escalating dose from 16 to 22 Gy. Tunel-positive cells were stained green and the nucleus was stained with DAPI at day 3.5 post-irradiation. Scale bar 50 μm

In order to limit bone marrow exposure, abdominal irradiation was performed [19]. The current standard endpoint for the study of acute GIS is animal lethality within 10 days of radiation exposure (LD50/10) (LD: lethal dose for 50% at 10 days). In this way, to define the sublethal dose, we administered an increasing dose up to 16 to 22 Gy to obtain a GIS characterised by a strong and rapid weight loss and death between 7 and 10 days. Exposure to 16, 18, 20 and 22 Gy was lethal in 0, 70, 75 and 100% of the mice within 14 days with a DL0/10, DL65/10, DL75/10 and DL100/10, respectively (Fig. 1a). Lethality was strongly correlated with weight loss, with about a 30% loss observed 7 days after irradiation in groups of animals irradiated at 18 and 20 Gy (Fig. 1b). The dose that induces a GIS was established on the basis of the number of regenerating crypts forming microcolonies 3.5 days after irradiation. It is a marker of intestinal integrity objectified by the presence of 10% of crypts per intestinal section with at least 8–10 cells per crypts and is correlated with survival. This time corresponds to the maximum expected difference between restoration and loss of crypts/villi. At day 3.5, histological analysis (HES) showed a change in tissue architecture observed from the 18-Gy dose with drastic reductions in villi length, hypoplasia of crypts and destruction of epithelial architecture (Fig. 1c). The analysis of the density of regenerating intestinal crypts was 66, 17, 8 and 1% at doses of 16, 18, 20 and 22 Gy, respectively, compared with non-irradiated mice (Fig. 1d). In parallel, representative images of TUNEL-positive cells characterising the irreversible cell death showed an increase in the frequency of TUNEL-positive cells in the mucosa with an escalating dose from 16 to 22 Gy compared with non-irradiated mice (Fig. 1e). Taken together, from the results of survival experiments and the histological analysis, a dose of 18 Gy was the sublethal dose inducing a GIS.

### SVF improves survival following a sublethal dose of irradiation and mitigates GIS

SVF was prepared from animal donors and characterised by flow cytometry. The gating strategy and subpopulation determination are shown in supplemental figure (S1). The viability of cells was >95%. Expression of mesenchymal, vascular, pericytic and haematopoietic markers as well their combinations showed that the percentage of the mesenchymal marker was >60% for CD90, CD44 and CD29; >50% for Sca-1; and >10% for CD105 and 0.3% for SSEA3. The SVF also contained haematopoietic cells with >60% CD45^+^CD3^+^, 2% CD45^+^CD11b^+^ and >20% CD45^+^CD11b^−^ B220^+^. Interestingly, SVF also contained a small amount (<1%) of endothelial mature cell (CD31^+^CD34^−^CD146^+^) and endothelial progenitor (CD31^+^CD34^+^CD146^+^).

**Fig. 2:**
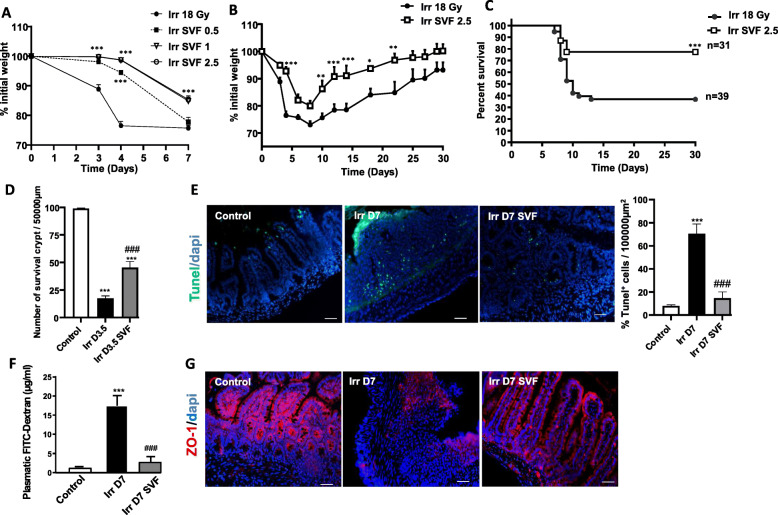
SVF treatment mitigating GIS. **a** Cell concentration-dependent effect of SVF on weight loss changes at 7 days of mice exposed to 18 Gy abdominal irradiation. **b** Weight loss changes for 30 days (n = 8) and **c** Kaplan-Meier survival analysis of 18 Gy abdominal exposed mice receiving SVF (2.5 × 10^6^ cells) treatment, or not receiving treatment. The data are represented by mean ± SEM; p values were calculated by ANOVA with Bonferroni correction; *p < 0.05; **p < 0.01; ***p < 0.001 compared with the irradiated group. **d** Number of regenerative crypts at day 3.5 after 18 Gy abdominal irradiation with or without SVF (2.5 × 10^6^ cells) treatment. **e** Representative immunostaining of Tunel and the number of Tunel^+^ cells in the ileal sections from the control in irradiated and irradiated SVF-treated mice 7 days post-irradiation. **f** Intestinal permeability checked by FITC/dextran assay in non-irradiated mice and 7 days after abdominal irradiation with or without SVF treatment. **g** Representative immunostaining of Zonulin-1 (ZO-1) in the ileal sections from the control in irradiated and irradiated SVF-treated mice 7 days post-irradiation. Scale bar 50 μm. The data are represented by mean ± SEM (n = 8). p values were calculated by ANOVA with Bonferroni correction; ***p < 0.001 compared with the control mice; ###p < 0.001 compared with the irradiated mice

Weight loss monitoring was first used to establish the concentration of SVF cells to be injected. At a concentration of 0.5 × 10^6^, SVF cells had no effect on weight loss on day 7 (about 25%) compared with the irradiated group. Seven days after irradiation, a loss of only 10% was observed in the groups of animals injected with 1 and 2.5 × 10^6^ (Fig. 2a). The SVF concentration of 2.5 × 10^6^ cells will be injected in future experiments. Treatment with SVF of 2.5 × 10^6^ cells limits weight loss and allows them to regain weight faster than the irradiated mice (Fig. 2b). In terms of mortality, while 65% of the untreated-irradiated mice died within 10 days, the SVF treatment saved 80% of mice from lethality (Fig. 2c).

High irradiation doses induce apoptosis of the crypt epithelial cells, resulting in a decrease in regenerating crypt colonies at 3.5 days and ultimately villi denudation by day 7 post-irradiation. The number of regenerative crypts microcolonies 3.5 days after irradiation was significantly increased in mice treated with SVF (44.5 ± 5.3/μm; p<0.001) compared with the irradiated mice (17.5 ± 2.2/μm) (Fig. 2d), indicating an intestinal regenerative response. Moreover, mice treated with SVF had a significant decrease in the number of Tunel^+^ cells (5-fold; p<0.001), especially near the position of the crypt cells 7 days after irradiation, compared with irradiated mice; the number and the localisation (far away from the crypt cells) of Tunel^+^ cells were similar to the non-irradiated mice (Fig. 2e).

Disruption of the intestine structural integrity alters its barrier function, making it permeable to luminal content. Dextran is unable to cross the intestinal epithelium when it is intact [20]. Intestinal permeability was quantified 7 days after irradiation with FITC-labelled dextran in the blood 4 h after gavage. Abdominal irradiation significantly increased intestinal permeability (17.5 ± 2.6μg/ml versus 1.4 ± 0.1μg/ml for the control; p<0.001). Treatment with SVF restores the impermeability (3.2±1.5 μg/ml) compared to the non-irradiated mice (Fig. 2f). The permeability was characterised by tight junction loss. Indeed, immunostaining of the tight junction protein zonula occludens-1 (ZO-1) showed that ZO-1 in the control group was expressed in the cytomembrane of epithelial cells along the villi. Seven days post-irradiation, the expression of ZO-1 was considerably reduced when SVF treatment restored its expression (Fig. 2g). These results indicate that SVF treatment induced a functional restoration of intestinal epithelial integrity.

### SVF increases intestinal regeneration

**Fig. 3:**
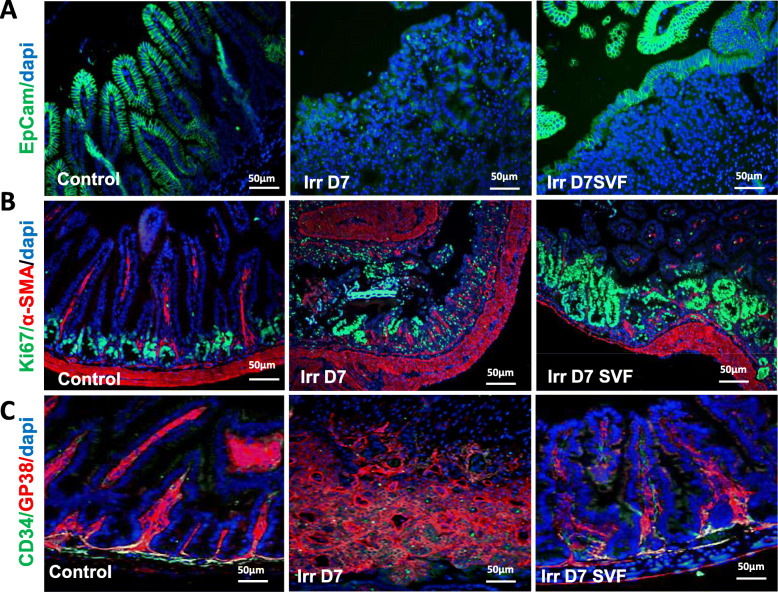
Ileal regenerative response induced by SVF treatment. Sections of ileum from mice 7 days after 18 Gy abdominal irradiation, with or without SVF treatment were stained with **a** EpCam (green) and **b** Ki67 (green). Note the hypertrophic (green) crypts under SVF treatment. **c** Co-staining of gp38 (red) and CD34 (green) identifying pericryptal mesenchymal cells. Double-positive gp38^+^CD34^+^ appeared in orange/yellow at the base of crypts. Dapi stained nuclei. Scale bars 50 μm

The maintenance of the epithelial barrier is also mediated by epithelial cell adhesion molecules such as EpCam, which also plays a role in cell proliferation, migration and differentiation [21]. In non-irradiated mice, EpCam expression is observed at the crypts and villi and localised on the plasma membrane. Seven days after irradiation, a decrease in EpCam expression was observed as well as a disturbance in its localisation, with a mislocalisation in cytoplasm (Fig. 3a). SVF treatment is associated with a recovery of EpCam expression both in the stem cell compartment and on the surface of the mucosa.

Decreased EpCam expression may also impact stem cell proliferation/differentiation [21]. In the control group, Ki-67 immunostaining showed that the majority of proliferating cells were located in the transient amplification cell compartment and did not reside in the base of the crypt. Irradiation leads to a decrease in the number of Ki-67^+^ cells in the ISC compartment. SVF treatment results in a significant increase in the number of Ki-67-positive cells in the ISC compartment, the transient amplification cell compartment and the extending regenerative epithelium, resulting in an enlarged and expanded hyperproliferative cluster area (Fig. 3b).

Mesenchymal-derived factors are essential for maintaining intestinal epithelial stem cell homeostasis. In particular, the double-positive stromal cells Gp38^+^CD34^+^ in the pericryptal localisation implicated in the production of niche factors are localised near the crypts in close contact with Lgr5^+^ stem cells [22]. The double immunostaining Gp38/CD34 showed that irradiation negatively impacts these stromal niches 7 days after irradiation and that the treatment by SVF enables them to be restored (Fig. 3c).

### SVF stimulates the restoration of the IECS

**Fig. 4:**
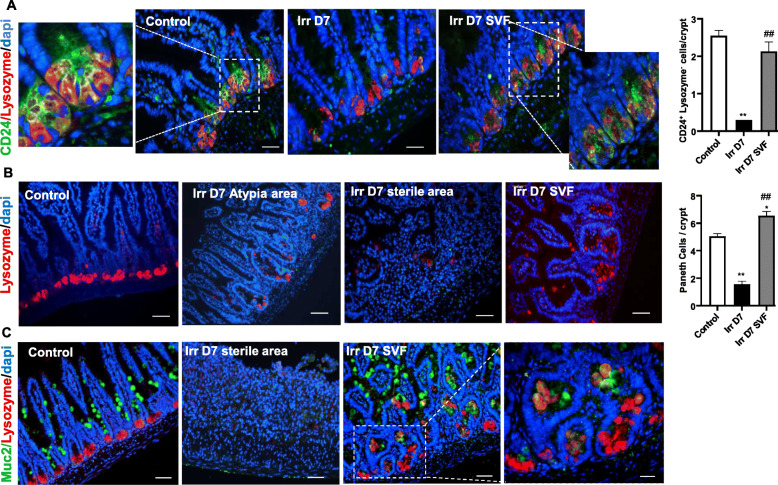
Effect of SVF on crypt IECS. **a** IHC staining showing the expression pattern of CD24 (green) and lysozyme (red) in the ileum. The cross section shows the co-staining of CD24 and lysozyme in the control and after SVF treatment. The histogram shows the restoration of the CD24 population after SVF treatment in crypts. **b** Lysozyme staining and histogram of the Paneth cell number per crypts. **c** IHC co-stained with lysozyme (red) and goblet cell marker Muc2 (green) to identify immature Paneth progenitor cells. Dapi stained nuclei. Scale bars 50 μm. The data are represented by mean ± SEM (n = 4–5). p values were calculated by ANOVA with Bonferroni correction, *p < 0.01; **p < 0.001 compared with the control group; ##p < 0.001 compared with the irradiated group

**Fig. 5:**
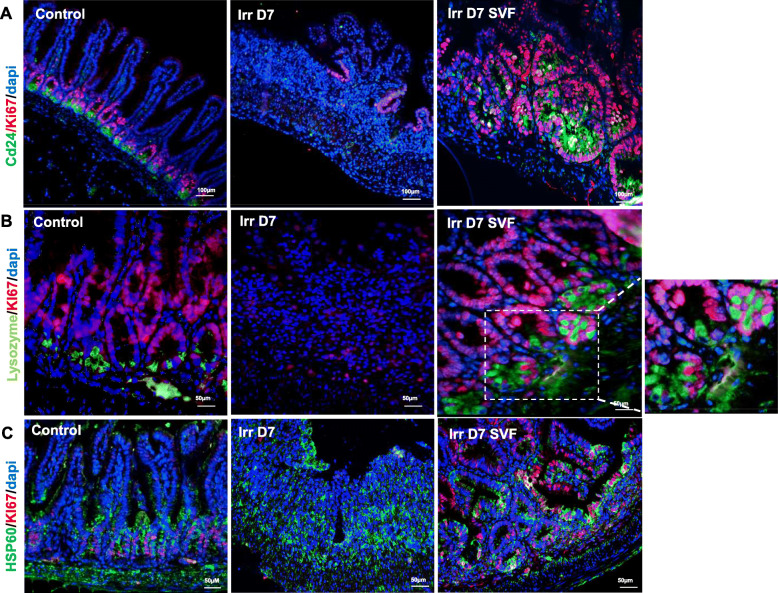
Crypt IECS proliferation induced by SVF treatment. **a** IHC co-staining CD24 (green) and Ki67 (red) in the ileum showing the hyperproliferative area after SVF treatment. **b** IHC co-staining lysozyme (green) and Ki67 (red) in the ileum. **c** IHC co-staining HSP60 (green) and Ki67 (red) in the ileum. Dapi stained nuclei

CD24 is a marker of the lower crypt IECS [23] and therefore used to identify LGR5^+^ and Paneth cells. In non-irradiated mice, we observed the presence of 2.5 CD24^+^ lysozyme^−^ cells per crypt, the corner-shaped cell population between Paneth cells (Fig. 4a). An 80% loss of these cells was observed at day 7 after irradiation and SVF treatment restored this cell population. Paneth cells contribute to intestinal homeostasis by synthesising peptides and antimicrobial proteins such as lysozyme and stem cell niche factors [2] and further promoting crypt base formation [24]. By using lysozyme staining to identify Paneth cells, we observed that the number of lysozyme^+^ cells decreased significantly on day 7 after irradiation (p<0.001) compared with the control mice (Fig. 4b). In the control mice, the lysozyme was normally efficiently packaged in well-defined secretory granules in the Paneth cells. In the irradiated mice, the Paneth cells exhibited altered lysozyme allocation patterns, with the majority showing reduced (lowly granular) or diffuse lysozyme staining. After SVF treatment, the number of Paneth cells per crypt increased compared with the irradiated mice (p<0.001), which was also higher than in the control mice. Interestingly, we also observed that the number of lysozyme^+^ cells in the “upper crypt” increased in mice treated with SVF, suggesting an aberrant ISC niche architecture. The co-localisation of MUC2 (goblet cell marker) and lysozyme (Paneth cell marker) staining characterised an “intermediate” goblet/Paneth cells and were definited as immature or bipotential progenitor cells [25]. Double immunostaining showed that the presence of Paneth cells at the “upper crypt” was co-staining with Muc2 after SVF treatment, indicating the presence of the “intermediate” cells (Fig. 4c). To assess whether the observed proliferative cluster was related to IECS, we performed a Ki67/CD24 co-staining. In the control mice, rare Ki67^+^/CD24^+^ cells were present in the crypts, with the majority of proliferating cells being located in the transit amplification compartment (TA) and not in the base of the crypt (Fig. 5a). On the other hand, in the proliferative cluster induced by SVF treatment, the CD24^+^ cells were Ki67^+^; an expansion of the TA zone was also observed. In addition, to determine which population was responsible for increased crypt cell numbers, a double Ki67/lysozyme staining was carried out and showed the presence of Ki67^+^/lysozyme^−^ and Ki67^+^/lysozyme^+^ cells 7 days after irradiation in the irradiated mice treated with SVF (Fig. 5b), indicating the proliferation of ISC and in particular the Paneth cells. It has recently been reported that hyperproliferative crypts show a high expression of HSP60, the mitochondrial molecular chaperone involved in the control of ISC homeostasis, and that HSP60-deficient crypts display a loss of stemness and cell proliferation, resulting in an aberrant Paneth cell phenotype [26]. Here, we observed an increase in the HSP60^+^ cells in the regeneration zone after treatment of SVF (Fig. 5c), indicating the absence of mitochondrial impairment. Together, these results showed that treatment with SVF restored the CD24^+^/lysozyme^−^ and Paneth cell populations and increased the proliferation of the TA compartment (Ki67^+^/CD24^+^) as well as the proliferation of Paneth cells (lysosyme^+^/Ki67^+^).

### SVF limits the inflammatory process by shifting the monocyte-macrophage phenotype

**Fig. 6:**
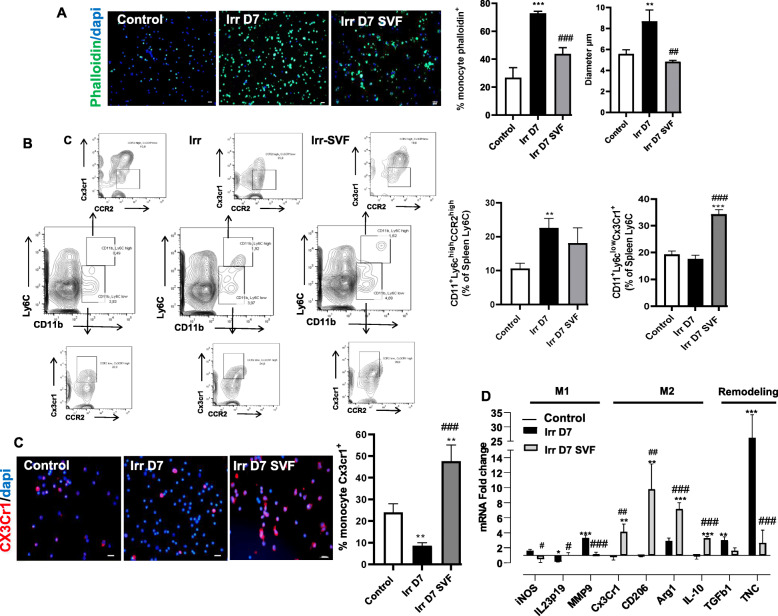
SVF promoting anti-inflammatory monocyte/macrophage subsets in the spleen. **a** Phalloidine staining (green) of splenic monocytes from the control and at day 7 post-irradiation with or without SVF treatment. Dapi stained nuclei. Scale bar 20 μm. The bar graphs are represented by the mean ± SEM of monocytes phalloidine^+^ and the monocyte diameters. **b** Representative FACS plots and frequencies of CD11b^+^Ly6C^high^CCR2^high^ and CD11b^+^Ly6C^low^Cx3cr1^high^ among live cells from the control and at day 7 post-irradiation with or without SVF treatment. **c** Cx3cr1staining (red) of splenic monocytes. The bar graphs are represented by the mean ± SEM of Cx3CR1^+^ monocytes. Dapi stained nuclei. Scale bar 20 μm. **d** Gene expression from splenic monocytes differentiated in different culture conditions from the control and at day 7 post-irradiation with or without SVF treatment. Macrophages were generated from splenic monocytes in the presence of M-CSF. Real-time PCR in an unstimulated condition, a M1 condition after stimulation with LPS and IFN-γ and a M2a condition after stimulation with IL-4. The data are expressed relative to control and normalised to GAPDH. All results are expressed as means ±SEM (n = 8). p values were calculated by ANOVA with Bonferroni correction, *p < 0.05; **p < 0.01; ***p < 0.001 compared with the control group; #p < 0.05; ##p < 0.01; ###p < 0.001 compared with the irradiated group

Macrophages have a central role in the epithelial barrier and in the maintenance of mucosal homeostasis. The spleen is the source of the main subsets of macrophage-generating monocytes [27, 28], and we therefore evaluated the percentage of pro-inflammatory and anti-inflammatory monocytes in the spleen 7 days after irradiation and the impact of SVF treatment on the subsets of splenic monocytes. Firstly, the migration potential of splenic monocytes was assessed using the spreading assay performed on a rmVCAM-1 matrix at day 7 after irradiation with and without SVF treatment. Unspread cells were defined as round cells, while spread cells were defined as cells with extended pseudopodia. The percentage of cells adopting the spreading morphology was quantified using fluorescein-conjugated phalloidin, which binds to filamentous actin (Fig. 6a). In the non-irradiated mice, actin filament staining was weak in the splenic monocytes and significantly increased in the irradiated mice (2.7-fold compared with the control; p<0.001). Treatment with SVF significantly reduced the intensity of phalloidin staining to 42% compared with the irradiated mice. This is associated with morphological changes characterised by a normalisation of cell diameter, suggesting an impact on the pro-inflammatory phenotype of these cells [29]. Flow cytometry analysis of pro-inflammatory and anti-inflammatory monocytes subsets showed a 2.5-fold (p<0.01) increase in the percentage of Ly6c^high^ pro-inflammatory monocytes defined as CD11b^+^Ly6c^high^CCR2^high^ in the irradiated mice compared with the control mice (Fig. 6b). Treatment with SVF did not reduce the percentage of Ly6c^high^ monocytes. On the contrary, the anti-inflammatory monocytes subset CD11b^+^Ly6c^low^CX3CR1^high^ of mice treated with SVF showed a 2-fold (p<0.001) higher percentage of anti-inflammatory Ly6c^low^ monocytes than the irradiated mice, which was confirmed by CX3CR1 immunostaining of the splenic monocytes (p<0.001) (Fig. 6c). In addition, the splenic monocytes could differentiate into pro-inflammatory M1 or anti-inflammatory M2 macrophages and contribute to tissue repair [30]. To address this issue, macrophages generated by 7 days of culture with M-CSF were stimulated with LPS-IFΝγ (M1 conversion) or IL-4 (M2 conversion) and the level of mRNA associated with macrophage polarity was analysed by real-time PCR. Our results showed that irradiation stimulated the expression of genes linked to the M1 marker (i.e., iNos, MMP9) without any effect on the expression of genes linked to the M2 marker (Fig. 6d). SVF treatment, on the other hand, reduced the mRNA levels of M1 (iNos, MMP9) markers and increased the mRNA levels of M2 markers (CX3CR1, CD206, Arg1, IL-10). Interestingly, the SVF treatment limited the levels of mRNA coding for excessive remodeling markers such as TGF-β 1 or TNC.

**Fig. 7:**
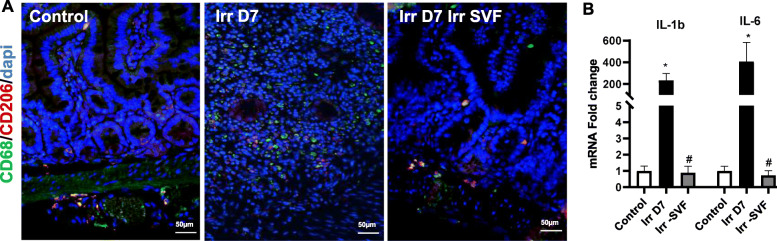
SVF facilitated induction of M2 macrophages in the ileum and reduced radiation-induced inflammatory response. **a** IHC co-staining CD68 (green) and CD206 (red) in the ileum showing an increase of the co-staining CD68/CD206 related to M2 macrophage after SVF treatment. Dapi stained nuclei. **b** Real-time PCR analysis of IL-1β and IL-6. The results are expressed as means ±SEM (n = 8). p values were calculated by analysis of variance with Bonferroni correction; *p < 0.001 compared with the control mice; #p < 0.001 compared with the irradiated mice

In the ileum, the double CD68/CD206 staining showed the absence of CD206^+^ cells in the irradiated mice and the SVF treatment led to an increase in CD68^+^/CD206^+^ cells (Fig. 7a). This anti-inflammatory effect in the intestine by the SVF treatment is supported by real-time PCR results with a significant suppression of mRNA levels coding for IL1-1β and IL-6 compared with the irradiated mice (p<0.001) (Fig. 7b). Taken together, these results suggest that SVF treatment changes the polarity of monocyte/macrophage to an anti-inflammatory phenotype, which may contribute to improving the GIS.

## Discussion

In the event of accidental or intentional exposure to high doses of radiation resulting in GI syndrome in many victims, there is an urgent need to implement effective and rapid therapeutic countermeasures. SVF can be produced within a few hours, injected on the same day and injected in an autologous way. In our study, we showed that intravenous injection of SVF limited the weight loss of the mice exposed to sublethal irradiation, inhibited the intestinal permeability which resulted in increased survival against lethal doses of radiation. Histological analyses showed that SVF treatment stimulated the regeneration of the epithelium by promoting numerous enlarged hyperproliferative zones and restored the cell populations in the ISC compartment. The therapeutic efficiency was associated to an anti-inflammatory effect.

Potential countermeasures to attenuate GIS have been identified by their ability to improve acute survival in mice (LD50/10) and to increase survival times at LD80/10 by at least 50% [19]. In our study, the therapeutic effect was very important in terms of mortality, with an 80% mortality in the irradiated and treated animals, compared to 38% in the untreated-irradiated animals. In addition to the practical advantages of obtaining SVF easily and quickly, the other advantage is its composition that is rich in different cell lineages, which are potentially pro-regenerating and therefore have more pleiotropic effects than MSCs [8–11, 31]. Indeed, previous studies have shown that the injection of MSC into irradiated mice resulted in improved regeneration of intestinal or colonic tissue, increasing the survival of the animals and reducing colonic ulcerations [10, 31]. The effectiveness of MSCs is due to the production of growth factors with anti-inflammatory and pro-angiogenic effects. The supportive effect of MSCs in the regeneration of the intestinal epithelium is not specific to this subpopulation of cells. However, in vitro amplification before their injection limits their use in clinic emergencies. Other cell types are involved in the regenerative response to irradiation such as endothelial cells and immune cells [32]. In our study, the characterisation of SVF in mice showed a high proportion of both MSCs and immune cells and the presence of endothelial progenitors. The combined presence of these different cell types seems to be responsible for the superiority of efficacy compared to the treatment of MSCs alone [33].

The mechanism of the therapeutic efficiency of SVF remains poorly understood. The literature supports the contribution of paracrine effects, with crosstalk between SVF components and the host, leading to repair and healing [12]. In our model, the SVF treatment both stimulated the epithelial cell regeneration and restored the impermeability as indicated by ZO-1 and EpCam IHC and measurement of permeability. Moreover, SVF treatment leads to hyperproliferative Ki67^+^ areas, features typical of actively regenerating intestinal epithelium [34] combined with the restoration of the Gp38^+^CD34^+^ mesenchymal cells associated to crypts, which plays a predominant role in the maintenance of ISCs pool [22, 25].

Under physiological conditions, ISCs LGR5^+^ located at the bottom of the crypts are responsible for the replenishment of intestinal epithelial cells. Using the genetic labelling and the tracing of distinct cell types, recent reports have indicated that Lgr5^+^ ISC are radiosensitive. At an abdominal dose of 10 Gy, the surviving Lgr5^+^ ISCs are still sufficient to sustain complete intestinal recovery 2 weeks after irradiation [35]; beyond this dose, the more widespread apoptosis of Lgr5^+^ ISCs induces a failure in the restoration of viability of the small intestine [36]. In our study, in order to visualise the ISC compartment, we used the co-location of CD24, more strongly expressed at the base of the crypt [23, 37, 38] and the lysozyme expressed by the Paneth cells that support the stem cell niche of the crypt [2]. We showed that irradiation caused a significant loss of ISC and Paneth cells. At day 7 after 18 Gy irradiation, the time and the dose where the modest surviving Lgr5^+^ ISCs could not dramatically support intestinal recovery, treatment with SVF restored the CD24^+^/lysozyme^−^ and Paneth cell populations, increased the proliferation of the TA compartment (Ki67^+^/CD24^+^) as well as the proliferation of Paneth cells (lysosyme^+^/KI67^+^), whereas under normal homeostasis, Paneth cells do not express ISC or proliferative markers in vivo [39].

It has already been shown that Paneth cells can return to the proliferative state and differentiate into all types of epithelial cells after radiation injury. Schmitt et al. [40] have indeed shown that, in acute inflammation, Paneth cells de-differentiate into proliferating stem-like cells that are able to form 3D organoids ex vivo (in the absence of LGR5^+^ cells), whereas LGR5^+^ cells alone cannot form organoids, and finally repopulate the epithelial wall in vivo [38]. In addition, a depletion of Paneth cells leads to a concomitant reduction of Lgr5^+^ ISCs [41]. In our work, SVF treatment was associated with a proliferation of Paneth cells as well as their number per crypt. Interestingly, this effect was also observed in the “upper crypt,” which could suggest a process of dedifferentiation [25]. Thus, we observed the presence of a lysozyme^+^/Muc2^+^ population after treatment with SVF. These cells expressed Paneth and goblet biomarkers reflecting a bipotential progenitor of secretory cells, or intermediate cells [25]. In addition, mitochondrial impairment reduced stemness induced by loss of Paneth cell functionality and ISC differentiation. Specifically, HSP60 deficiency in ISC has been associated with complete loss of proliferation [26]. SVF treatment restored a mitochondrial function in the proliferation area which may have ensured the stemness.

Inflammatory cells such as monocytes/macrophages influence intestinal epithelium repair and generate dysfunctional PCs characterised by loss of lysozyme positive granules, concomitant to aberrant ISC phenotype such as observed in active ileal Crohn’s disease [26, 42]. Intestinal mucosa macrophages are dependent on constant replenishment by classical blood monocytes [43] and the spleen produces a majority of monocyte subsets generating macrophages [28]. Tissue repair after inflammatory insult requires coordinated mobilisation of both subsets: Ly6C^high^ monocytes, which digest damaged tissue, but their persistence is deleterious and Ly6C^low^ monocytes act on wound healing [44]. The spleen is the reservoir accommodating the demands of rapid-onset inflammation and we showed that the spleens from the irradiated mice were characterised by an accumulation of pro-inflammatory monocyte subset Ly6C^high^CCR2^high^ phenotypes and a high potential of migration characterised in the spreading assay. In the spleens from the SVF-treated mice, although the pro-inflammatory monocytes level was not reduced, we inversely observed an increase in the level of anti-inflammatory monocytes Ly6C^low^ Cx3cr1^high^, which can be matured in vitro in the anti-inflammatory macrophage M2. Our results support the fact that SVF treatment is associated with anti-inflammatory process both by repressing the pro-inflammatory cytokines and by shifting the macrophage polarity toward the M2 phenotype implicated in the inflammatory resolution phase and tissue regeneration [42]. More experiments are needed to understand the role of M2 macrophages in the ISC compartment after SVF treatment. Recently, it was shown that the depletion of macrophages resulted in decreased as well as crypt fission, Lgr5^+^ stem cell proliferation and Paneth cell numbers [45].

## Conclusion

In this work, we have shown that treatment with SVF mitigates the GIS. This cell therapy product stimulates regeneration and intestinal repair targeting the stem cell compartment and provides an immunomodulatory response that enables the animals to survive at lethal doses.

## Supplementary Information

**Additional file 1: Figure S1.** Gating strategies utilized for analyzing sub-populations within the stromal vascular fraction of mice. a) ASC-like populations. Isotype controls are shown as blue histogram. b) Leucocytes populations.

**Additional file 2: Table S1.** Taqman primers and probes.

## Data Availability

All relevant data and material to reproduce the findings are available in the manuscript.

## Abbreviations

SVF: Stromal vascular fraction
GIS: Gastrointestinal syndrome
Gy: Gray
ISCs: Intestinal stem cells
EpCam: Epithelial cell adhesion molecule
IECS: Intestinal epithelial stem cells
