# Supplementary figures and images for: The stromal vascular fraction mitigates radiation-induced gastrointestinal syndrome in mice
Source: Stem Cell Res Ther. 2021 May 29;12:309. doi: 10.1186/s13287-021-02373-y (PMC8164266; doi:10.1186/s13287-021-02373-y)

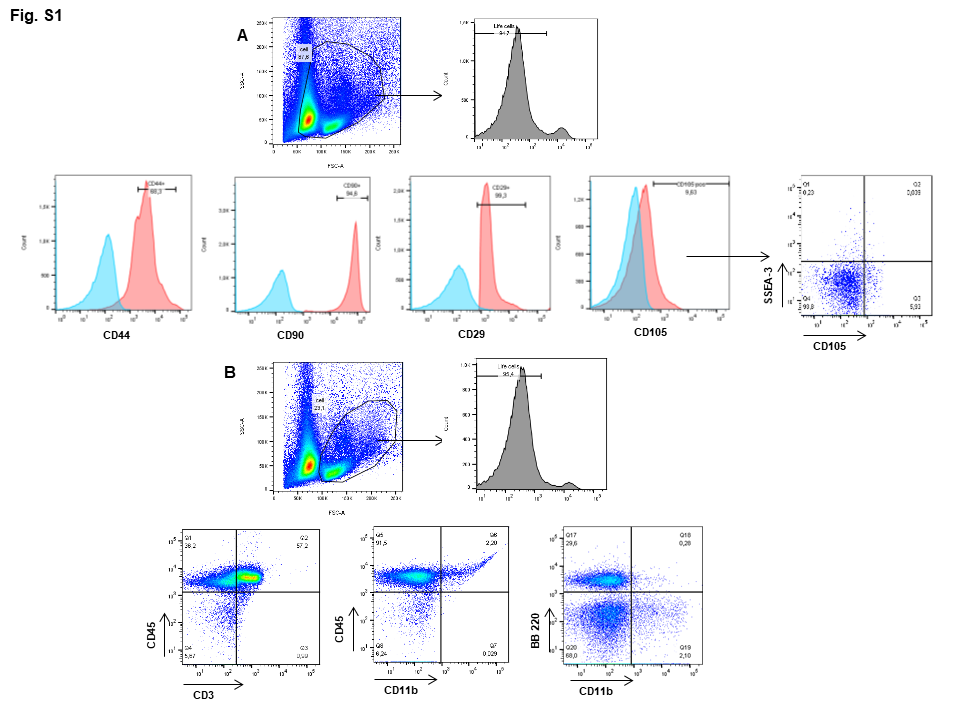

Supplement: Supplementary file 1 — Additional file 1: Figure S1. Gating strategies utilized for analyzing sub-populations within the stromal vascular fraction of mice. a) ASC-like populations. Isotype controls are shown as blue histogram. b) Leucocytes populations. [file 13287_2021_2373_MOESM1_ESM.tif]
