# Supplementary material for: The stromal vascular fraction mitigates radiation-induced gastrointestinal syndrome in mice
Source: Stem Cell Res Ther. 2021 May 29;12:309. doi: 10.1186/s13287-021-02373-y (PMC8164266; doi:10.1186/s13287-021-02373-y)
Supplement: Supplementary file 2 — Additional file 2: Table S1. Taqman primers and probes. [file 13287_2021_2373_MOESM2_ESM.pdf]

Table1.

Gene	TaqMan assay
IL-1β	Mm00434228_m1
IL-6	Mm00446190_m1
IL23p19	Mm00518984_m1
IL-10	Mm01288386_m1
Inos (Nos2)	Mm00440502_m1
CX3Cr1	Mm00438354_m1
Cd206 (Mrc1)	Mm01329362_m1
Arg1	Mm00475988_m1
MMP9	Mm00442991_m1
Tgf-β1	Mm01178820_m1
Tenacin C (TNC)	Mm00495662_m1
